# Construction of immune score and its prognostic value in invasive lobular carcinoma of the breast using computational pathology analysis

**DOI:** 10.1002/cam4.6896

**Published:** 2023-12-27

**Authors:** Liye Wang, Peng Sun, Fei Xu, Qiufan Zheng, Kuikui Jiang, Ruoxi Hong, Shusen Wang

**Affiliations:** ^1^ Department of Medical Oncology Sun Yat‐Sen University Cancer Center, State Key Laboratory of Oncology in South China, Collaborative Innovation Center for Cancer Medicine Guangzhou Guangdong China; ^2^ Department of Oncology the First Affiliated Hospital of Zhengzhou University Zhengzhou Henan China

**Keywords:** computational pathology analysis, immune score, invasive lobular breast carcinoma, prognosis, tumor‐immune microenvironment

## Abstract

**Background:**

Previous studies have shown that high level of TILs in invasive lobular carcinoma (ILC) is associated with poor prognosis, contrary to that in TNBC and HER2‐positive breast cancer.

**Methods:**

The densities of six immune cell markers and three immune checkpoints in the ILC microenvironment were detected by computational pathology analysis. Then, the LASSO cox regression model was used to construct an immune score (IS) and further evaluate its prognostic value.

**Results:**

In our ILC cohort, the low density of CD4, CD8, CD20, CD56, CD68, FOXP3, PD‐1, and PD‐L1 had significantly longer disease‐free survival (DFS) and overall survival (OS); however, the low density of CTLA‐4 was associated with shorter DFS and OS. Based on this, an IS was constructed, and patients with low‐IS had significantly prolonged DFS (*p* < 0.0001) and OS (*p* < 0.0001). Multivariate analysis revealed that IS was an independent prognostic indicator for DFS and OS. Further analysis showed that IS may increase the prognostic value of TNM stage. We further explored the prognostic role of CD68 and FOXP3 in the transcriptional level and the corresponding ISm in the METABRIC dataset, and found that low proportion of CD68 and FOXP3 and their ISm were associated with longer OS, and ISm was also an independent prognostic factor for OS.

**Conclusion:**

IS was a promising biomarker to distinguish the prognosis in ILC patients.

## 1 BACKGROUND

Invasive lobular carcinoma (ILC) accounting for 5%–15% of all breast cancer cases.^1^ ILC is characterized by hormone receptor (HR)‐positive with a low proliferation rate.^1^ Conventionally, tumor/lymph node/metastasis (TNM) staging has been used to the main tool in determining treatment strategy and assessment of prognosis. However, this anatomy‐based system provides the clinical prognosis may vary significantly among ILC patients with the same TNM stage receive similar treatment, due to its highly heterogeneous nature.^2^ Thus, researchers are intensely searching for complementary factors such as tumor‐intrinsic genetic features^3^ or extrinsic immune factors.^4^ Important studies have found the major role played by the immune response in ILC biology.^2^, ^5^ Tumor‐infiltrating immune cells and high expression of immune checkpoints by tumor or immune cells are key components of the adaptive immune system with a crucial impact on cancer progression.^6^ This indicates that immune markers might play an important role in the development of ILC. However, the role of immune factors as prognostic biomarkers in the tumor microenvironment in patients with ILC remains unclear.

The tumor‐immune microenvironment encompasses the surrounding immune cells, lymphocytes, and bone marrow‐derived inflammatory cells.^7^ These cell types include effector CD8+ and CD4+ T cells, B cells, naive and memory lymphocytes, macrophages, natural killer (NK) cells, dendritic cells, mast cells, and other immune cell subtypes.^8^ In HR‐negative invasive breast tumors, higher quantity of tumor‐infiltrating lymphocytes (TILs) has strong prognostic associations, particularly in the setting of early‐stage triple‐negative breast carcinomas (TNBC)^9^ and human epidermal growth factor receptor 2 (HER2)—positive breast carcinomas^10^ treated with adjuvant chemotherapy, while TILs represent a promising new morphologic biomarker associated with poor outcome of ILC.^4^ However, due to the visual assessment of the density of TILs in tumor tissue stained with hematoxylin and eosin is unreliable and has limitations when applied to large‐scale populations. Quantification of immune markers using computational pathology and construction of comprehensive immune score (IS) system has been shown to make more accurate prognoses for some cancer types.^11^, ^12^, ^13^ However, an IS system with prognostic significance for ILC patients in clinical settings has not been identified so far.

In recent years, a hallmark of tumor progression is the tumor immune evasion,^14^ which plays a major role in modulating innate immune and suppressing T‐cells,^15^ leading to tumor growth and progression. Immune checkpoints are the most important signaling pathways mediating tumor immune escape, which are crucial for modulating duration and amplitude of immune response in peripheral tissues and maintaining autoimmune tolerance.^16^ The main immune checkpoints for breast cancer include programmed death receptor 1/programmed cell death ligand 1 (PD‐1/L1), cytotoxic T‐lymphocyte‐associated protein‐4 (CTLA‐4), and other molecules. However, the prognostic value of PD‐L1/PD‐1 and CTLA‐4 in ILC and their interactions with different immune cells remain unclear.

In the current study, we evaluated the density of immune cells (CD4, CD8, CD20, CD56, CD68, and FOXP3) and immune checkpoints (PD‐1, PD‐L1, and CTLA‐4) for ILC patients using computational pathology. We then explored the prognostic values of these immune biomarkers. In addition, we developed an IS based on multivariate analysis of immune cells to predict clinical outcomes of ILC patients. This approach can divide ILC patients into different risk subgroups and might add prognostic value to the TNM staging system. We further explored the prognostic value of CD68 and FOXP3 and their ISm with the transcriptome data of ILC patients in the METABRIC database.

## 2 METHODS

### 2.1 Patients and database

We retrospectively collected formalin‐fixed paraffin‐embedded (FFPE) tumor specimens and clinical data from 172 nonmetastatic ILC patients between May 2003 and December 2017 in our hospital. No patients had received any antitumor therapy before surgery, and all of the patients were pathologically diagnosed with ILC. All patients were restaged according to the 8th AJCC TNM staging system.^17^ The METABRIC (Molecular Taxonomy of Breast Cancer International Consortium) dataset^18^ containing 1904 tumor cases was downloaded from the cBioPortal database (http://www.cbioportal.org/) (access date: November 30, 2021). A total of 139 ILC samples with full clinical characteristics and transcriptome data and performed the following data exploration. The study has been approved by the ethical committee of Sun Yat‐Sen University Cancer Center (Ethics approval number of clinical study project: B2021‐061). All the patients provided written informed consent prior to inclusion into the study.

### 2.2 Immunohistochemistry (IHC)

Sequential histological tumor sections of 4 μm thick were obtained from a representative FFPE tumor block and used for IHC analysis. The IHC assay was performed as previously described.^19^ IHC staining was performed for: six immune cells (CD4^+^ helper T cells, CD8^+^ cytotoxic T cells, CD20^+^ B cells, CD56^+^ NK cells, CD68^+^ total macrophages, and FOXP3^+^ regulatory T cells) and three immune checkpoints (PD‐1, PD‐L1, and CTLA‐4). The following primary antibodies were used: anti‐CD4 (ZA‐0519, 1:200; ZS), anti‐CD8 (ZA‐0508, 1:200; ZS), anti‐CD20 (kit‐0001; MXB), anti‐CD56 (ZM‐0057, 1:200; ZS), anti‐CD68 (ZM‐0060, 1:800; ZS), anti‐FOXP3 (UM800140, 1:100; ZS, Ultra‐MAB), anti‐PD‐1 (ZM‐0381, 1:200; ZS), anti‐PD‐L1 (clone E1L3N, 1:100 dilution; Cell Signaling Technology, USA), and anti‐CTLA‐4 (SP355, 1:100; Abcam).

### 2.3 Computational pathology analysis

All IHC slides were examined by two independent pathologists, only slides with good staining quality would be included in image acquisition. The pathologists score immune markers according to a five‐step standardized scoring system developed by the International Immuno‐oncology Biomarker Working Group.^20^, ^21^ The quantification of immune markers in the entire studied area was assessed within the invasive tumor's borders.^21^ In addition, all normal and necrosis tissues were excluded from the assessment through manual annotation by pathologists. Then, a full view of each IHC slide was digitally scanned at 20× magnification, with an image resolution of 0.25 μm/pixel (Aperio, ScanScope AT2, Leica). All images were autoexamined using computational pathology analysis, and the expression was quantified on tumor cells (TCs) or tumor‐associated immune cells (TAICs) expressing the immune checkpoints. Representative IHC staining images with high and low positive numbers for these immune markers are shown in Figure S1.

The computational pathology analysis was performed as previously described and showed good performance,^21^, ^22^ and the methods are briefly described here. First, the individual cell nuclei were manual annotated as a TC or TAIC by two independent pathologists. Then, each nucleus in the hematoxylin channel was segmented using stain deconvolution and achieved good performance.^11^, ^23^ Computational pathology analysis can automatically segment of the nuclei in the hematoxylin channel, and automatically classify of the cells into TCs or TAICs based on Xception deep learning model.^24^ The computational pathology analysis showed a high reproducibility and consistency with pathological classification. Finally, the density of TCs or TAICs could be quantified as the total number of TCs or TAICs divided by the entire nonnecrotic area.

### 2.4 Construction of the immune score (IS)

We adopted a least absolute shrinkage and selection operator (LASSO) Cox regression model^25^ to select the most useful prognostic features out of all six immune cell markers and then constructed an IS for predicting survival. The analysis was performed by using the “glmnet” package in R software.^26^ Ten‐time cross validations with the Lambda.min criteria were used to determine the optimal values of λ, and a value of λ = 0.047 with log (λ) = −3.121 was chosen. Based on this value, CD4, CD20, CD68, and FOXP3 were selected. Among four immune cell markers, the CD68 and FOXP3 were identified significantly affecting OS and DFS and used to construct an IS by multivariate Cox regression analysis.

The optimal cutoff threshold immune cell density was used to stratify patients into groups based on the degree of tumor infiltration. This method has been independently validated in evaluating IS in multiple solid tumors,^11^, ^13^, ^27^ and was thus adopted for the current study. We then used X‐tile software (version 3.6.1; Yale University, New Haven, CT, USA)^28^ to determine the optimal cutoff values for high and low density regarding CD68 and FOXP3 based on the associations with patient disease‐free survival (DFS). Based on the threshold, each patient was given a binary score (0 for low and 1 for high) for each immune cell type (CD68 and FOXP3). IS for each patient was obtained by adding the two binary score values, the scale being from 0 to 2. Three patient groups were defined: patients with low densities of CD68 and FOXP3 were classified as IS‐0; patients with one high density for one marker were classified as IS‐1; and patients with two high densities of these two markers were stratified as IS‐2. Patients with a high degree of immune cell infiltration (IS: 2) were assigned as the high‐IS group, and patients with a low degree of immune cell infiltration (IS: 0–1) were assigned as the low‐IS group.

### 2.5 Construction of the immune score in the METABRIC dataset (ISm)

To identify immune characteristics of 139 ILC samples in the METABRIC dataset, their expression data were imported into CIBERSORT (https://cibersort.stanford.edu/) and iterated 1000 times to estimate the relative proportion of 22 types of immune cells. The 22 types of infiltrating immune cells inferred by CIBERSORT include B cells, T cells, natural killer cells, macrophages, dendritic cells, eosinophils, and neutrophils. The relative proportions of the same immune markers were added together to obtain the total ratio. The CD68^+^ total macrophages include M0, M1, and M2 macrophages. We used X‐tile software to determine optimal cutoff values for high and low proportion regarding CD68 and FOXP3 cells for OS.

We further explored the prognostic value of immune cell markers CD68 and FOXP3 and their ISm constructed from this dataset. According to the IS grouping method outlined above (0 for low proportion and 1 for high proportion), ILC patients were also divided into three groups: ISm‐0, ISm‐1, and ISm‐2. Patients with a high degree of immune cell infiltration (ISm: 2) were assigned as the high‐ISm group, and patients with a low degree of immune cell infiltration (ISm: 0–1) were assigned as the low‐ISm group. Then, we compared the relative proportions of 22 types of immune cells in patients with different clinicopathological factors and ISm subgroups.

### 2.6 Statistical analyses

Data are presented as whole numbers and proportions for categorical variables, and medians or means (interquartile ranges (IQRs)) for continuous variables. Clinicopathological variables associated with IS were analyzed using the chi‐square test or Fisher's exact test. Differences between groups were assessed using the Student *t*‐test or Mann–Whitney *U* test for continuous variables. DFS was calculated from the date of surgery to the date of disease relapse (local or distant relapse or death from any cause). Overall survival (OS) was calculated from the date of surgery to the date of death or the latest follow‐up. The Kaplan–Meier method was used to estimate OS and DFS and differences were compared using the log‐rank test. Calculating the exponential of the regression coefficients from the Cox model provided an estimate of the hazard ratios (HRs) and the 95% confidence interval (CI). ROC curves were used to compare IS prognostic validity with immune checkpoints, and to compare the prognostic validity of IS in different molecular subtypes. Multivariate Cox regression analysis with backward selection was performed to test the independent significance of different factors. Multivariate analysis was performed using variables with *p* < 0.1 in the univariate analysis, and only independent prognostic factors were retained in the multivariate model. In addition, we established a prognostic score model combining the IS, ER, and TNM stage. Moreover, nomograms predicting 3 years or 5 years OS were established. The model performance was evaluated by the accuracy of point estimates of the survival function (calibration). The performance of the nomograms was evaluated using the concordance index (C‐index).^29^ In addition, bootstraps with 1000 resamples were applied to internal validation to provide an unbiased estimate of model performance.

Statistical analyses were performed with software programs (SPSS version 26.0 (IBM); R version 4.1.2; GraphPad Prism 8). All statistical tests were two‐sided and considered significant when the *p* value was less than 0.05.

## 3 RESULTS

### 3.1 Patient characteristics and immune markers

The clinicopathological characteristics of ILC patients are summarized in Table 1. The majority of patients were HR‐positive: Luminal‐A (40.1%, *n* = 69) and Luminal‐B (54.7%, *n* = 94), only 2.3% were HER2‐positive and 2.9% were TNBC. Together, 48.8% patients had lymphatic invasion, and 29.1% patients belonged to stage III according to the criteria released by the 8th AJCC. The median follow‐up time was 84.6 months (range: 13.8–224.4 months). At the end of follow‐up, 34 (19.8%) patients had disease progression and 25 (14.5%) patients had died from ILC.

**TABLE 1:** Clinicopathological characteristics of the patients stratified by immune score.

	All (*n*%)	Low (*n*%)	High (*n*%)	*p* Value
Total population	172 (100)	149 (86.6)	23 (13.4)	
Age				0.653
≤ 50 years	99 (57.6)	87 (58.4)	12 (52.2)	
> 50 years	73 (42.4)	62 (41.6)	11 (47.8)	
Menopausal status				0.819
Premenopausal	109 (63.4)	95 (63.8)	14 (60.9)	
Postmenopausal	63 (36.6)	54 (36.2)	9 (39.1)	
T stage				0.001
T1	83 (48.3)	79 (53.0)	4 (17.4)	
T2‐T3	89 (51.7)	70 (47.0)	19 (82.6)	
N stage				0.001
N0	88 (51.2)	83 (55.7)	5 (21.7)	
N1	36 (20.9)	32 (21.5)	4 (17.4)	
N2‐N3	48 (27.9)	34 (22.8)	14 (60.9)	
TNM stage				< 0.0001
I	54 (31.4)	52 (34.9)	2 (8.7)	
II	68 (39.5)	62 (41.6)	6 (26.1)	
III	50 (29.1)	35 (23.5)	15 (65.2)	
ER expression				0.625
Yes	162 (94.2)	141 (94.6)	21 (91.3)	
No	10 (5.8)	8 (5.4)	2 (8.7)	
PR expression				0.517
Yes	149 (86.6)	130 (87.2)	19 (82.6)	
No	23 (13.4)	19 (12.8)	4 (17.4)	
HER2 expression				1.000
Yes	14 (8.1)	12 (8.1)	2 (8.7)	
No	158 (91.9)	137 (91.9)	21 (91.3)	
KI67				0.072
≤ 14	92 (53.5)	84 (56.4)	8 (34.8)	
> 14	80 (46.5)	65 (43.6)	15 (65.2)	
Subtype (based on receptor status)				0.069
Luminal A	69 (40.1)	64 (43.0)	5 (21.7)	
Luminal B	94 (54.7)	78 (52.3)	16 (69.6)	
HER2‐positive	4 (2.3)	4 (2.7)	0	
TNBC	5 (2.9)	3 (2.0)	2 (8.7)	
Chemotherapy				0.150
Yes	116 (67.4)	97 (65.1)	19 (82.6)	
No	56 (32.6)	52 (34.9)	4 (17.4)	
Radiotherapy				0.336
Yes	52 (30.2)	43 (28.9)	9 (39.1)	
No	120 (69.8)	106 (71.1)	14 (60.9)	
Endocrine therapy				0.453
Yes	156 (90.7)	136 (91.3)	20 (87.0)	
No	16 (9.3)	13 (8.7)	3 (13.0)	
Death				< 0.0001
Yes	25 (14.5)	15 (10.1)	10 (43.5)	
No	147 (85.5)	134 (89.9)	13 (56.5)	
Disease progression				0.001
Yes	34 (19.8)	23 (15.4)	11 (47.8)	
No	138 (80.2)	126 (84.6)	12 (52.2)	

Abbreviations: ER, estrogen receptor; HER2, human epidermal growth factor receptor 2; TNM, tumor‐node‐metastasis; PR, progesterone receptor.

**FIGURE 1:**
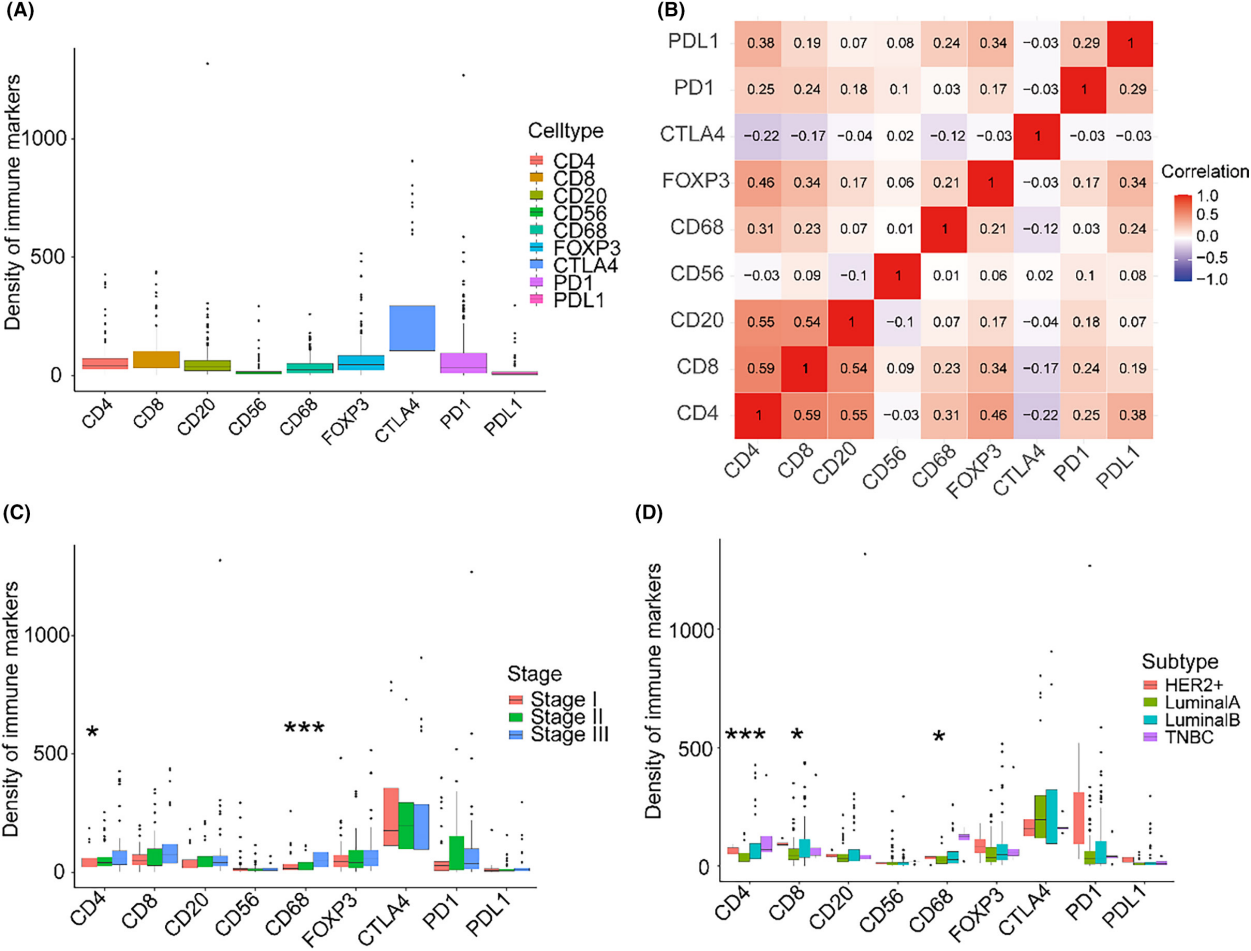
Composition and distribution of tumor‐associated immune markers in ILC. Composition of tumor‐associated immune markers (A) and correlation between immune markers (B) in ILC. Distribution of infiltrating immune cell markers and immune checkpoints density according to tumor stage (C) and different molecular subtypes (D). The thick lines represent the median value. The bottom and top of the boxes are the 25th and 75th percentiles (interquartile range), respectively. The scattered dots represent the corresponding subgroups in the graph. Significant statistical differences between the two subgroups were assessed using the Mann–Whitney test (*p < 0.05, *** *p* < 0.001).

Based on computational pathology analysis, we investigated the numeration of six immune cell markers and three immune checkpoints per mm^2^. Next, we analyzed the distribution of immune markers and their correlations (Figure 1). We observed that the CTLA‐4 expression (median density: 175.07 cells/mm^2^) was the highest among immune markers (Figure 1A). We found that CD4^+^ T cells was strongly correlated with CD8^+^ T cells and CD20^+^ B cells, while CTLA‐4 expression was negatively correlated with immune cells and other immune checkpoints except for CD56^+^ NK cells (Figure 1B). We also observed that CD4^+^ T cells and CD68^+^ macrophages were upregulated in the stage III group, while CTLA‐4 and PD‐1 expression were upregulated in the stage II group (Figure 1C). Meanwhile, we found that CD4^+^ T cells and CD68^+^ macrophages were upregulated in the TNBC subtype, while CD8^+^ T cells was upregulated in the HER2‐enriched subtype (Figure 1D).

### 3.2 Prognostic value of each biomarker

**FIGURE 2:**
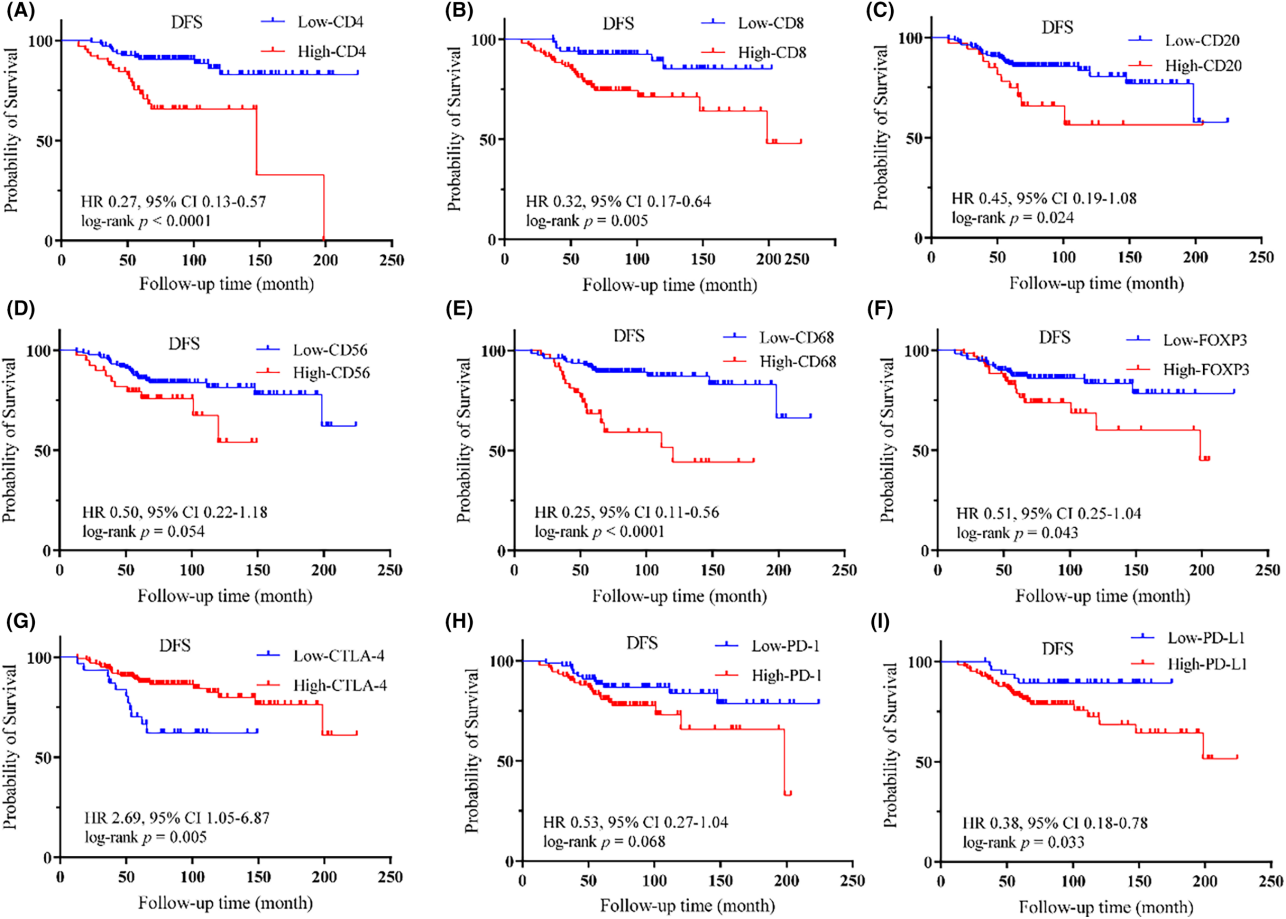
Kaplan–Meier curves for disease‐free survival according to different immune markers groups. Plots show the Kaplan–Meier curves of CD4 (A), CD8 (B), CD20 (C), CD56 (D), CD68 (E), FOXP3 (F), CTLA‐4 (G), PD‐1 (H) and PD‐L1 (I).

We then explored the prognostic value of each marker. X‐tile was used to determine optimal cutoff values for high and low density regarding six immune cell markers and three immune checkpoints for DFS (Table S1). As shown in Figure 2, patients with low‐infiltrating CD4^+^ T cells (*p* < 0.0001), CD8^+^ T cells (*p* = 0.005), CD20^+^ B cells (*p* = 0.024), CD68^+^ macrophages (*p* < 0.0001), or FOXP3^+^ T cells (*p* = 0.043) had better DFS than patients with high‐infiltrating. The patients with low expression of PD‐L1 (*p* = 0.033) was associated with better DFS than high expression, while low expression of CTLA‐4 (*p* = 0.005) was associated with poorer DFS than high expression (Figure 2). The associations between the nine immune markers and OS are shown in Figure S2.

### 3.3 IS and association with prognosis

**FIGURE 3:**
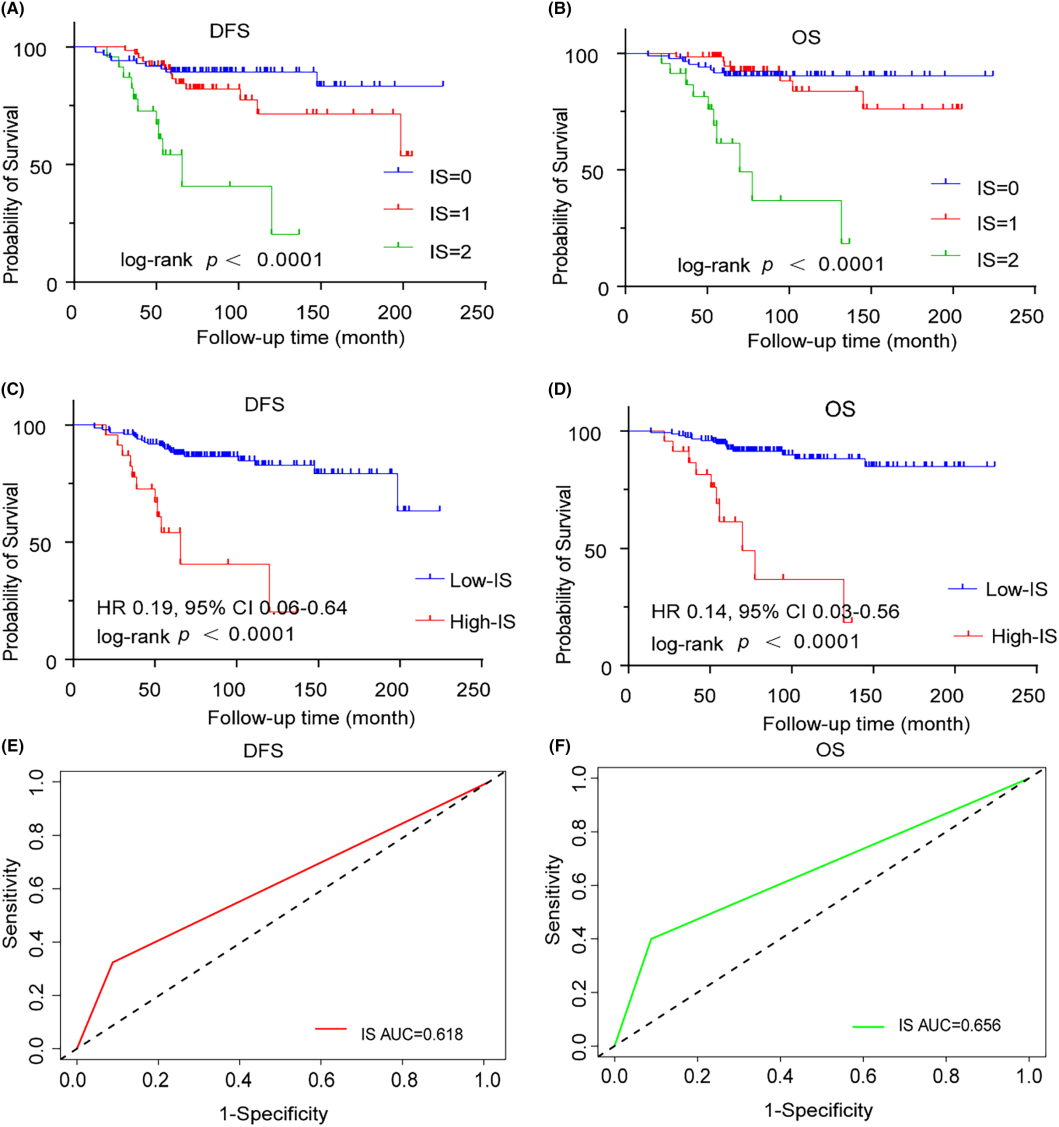
Kaplan–Meier curves for DFS and OS according to the immune score. Kaplan–Meier curves comparing DFS (A) and OS (B) in patients with different IS subgroups. Patients were stratified in high and low IS groups using the optimal cutoff value. Kaplan–Meier curves comparing DFS (C) and OS (D) in patients with high and low IS groups. Receiver operating characteristics (ROC) curves for the prediction of DFS (E) and OS (F).

To construct an IS, we identified two immune cell markers CD68 and FOXP3 that were significantly associated with DFS using penalized LASSO Cox regression models (Figure S3). According to the definition of IS (see Section 2), 49.4%, 37.2%, and 13.4% of the patients were classified as IS‐0, IS‐1, and IS‐2, respectively. We aimed to explore the prognostic value of IS. The Kaplan–Meier curves revealed three distinct patient groups with statistically significant differences in DFS (*p* < 0.0001, Figure 3A) and OS (*p* < 0.0001, Figure 3B). Patients with an IS‐0 experienced the best postoperative outcome, while patients with an IS‐2 had the worst outcome.

Patients with a high degree of immune cell infiltration (IS: 2) were assigned as the high‐IS group, and patients with a low degree of immune cell infiltration (IS: 0–1) were assigned as the low‐IS group. Based on this, we assigned 149 (86.6%) patients into the low‐IS group and 23 (13.4%) patients into the high‐IS group. Patients with low‐IS had longer DFS (*p* < 0.0001) and OS (*p* < 0.0001) compared with patients with high‐IS (Figure 3C, D). IS could be as a prognostic factor for DFS (AUC = 0.618) and OS (AUC = 0.656) in the ILC cohort (Figure 3E, F).

**FIGURE 4:**
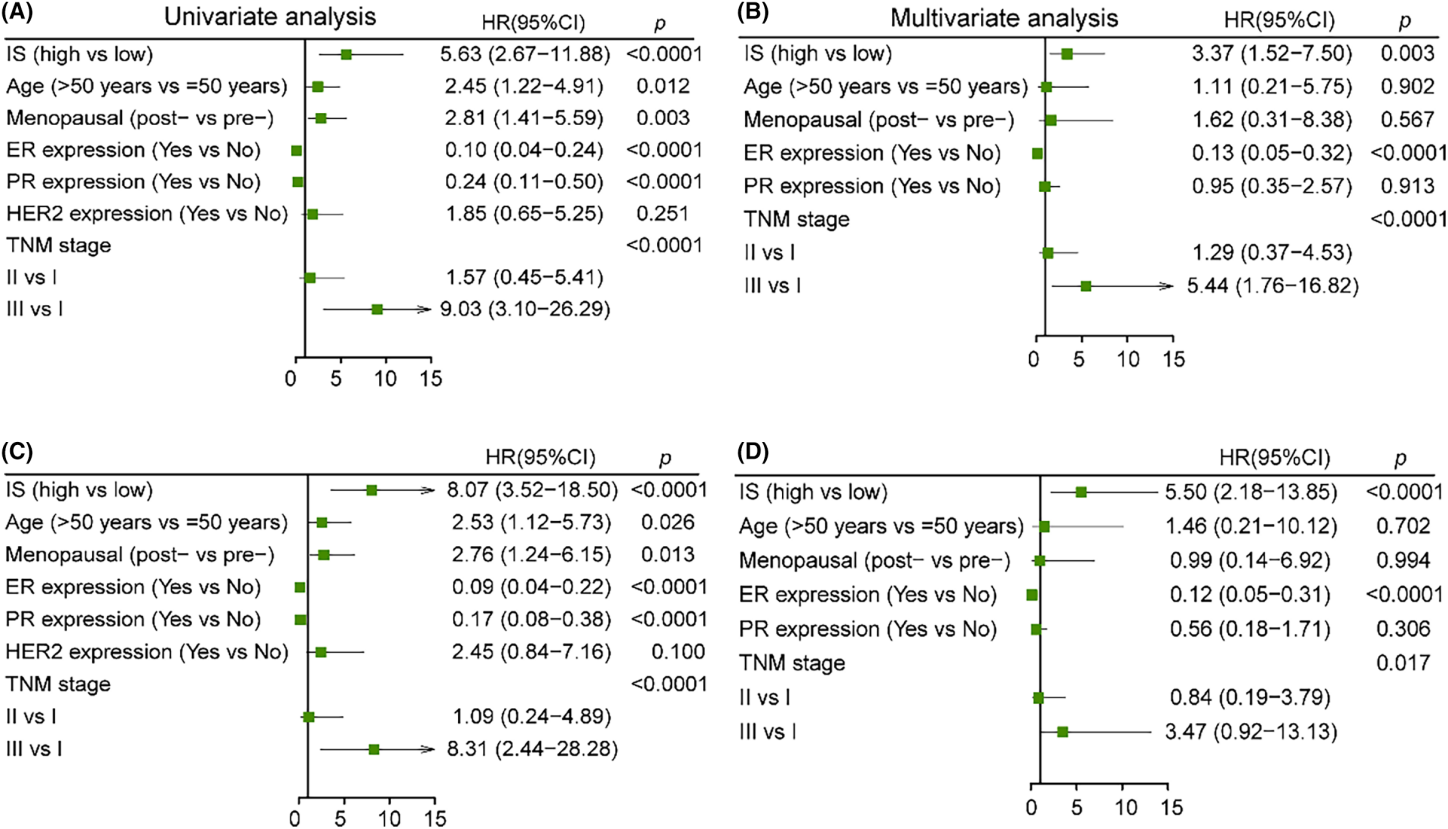
Univariate and multivariate analysis of factors associated with DFS and OS. Plots show univariate (A) and multivariate (B) analysis of DFS; univariate (C) and multivariate (D) analysis of OS.

We performed univariate analysis showed that IS was significantly associated with DFS (Figure 4A) and OS (Figure 4C). We then performed multivariate Cox regression analysis, which showed that the IS remained significant for DFS (*p* = 0.003 Figure 4B) and OS (*p* < 0.0001 Figure 4D). In addition, the TNM stage and ER levels were also significantly associated with DFS and OS in multivariate analysis (Figure 4B, D).

### 3.4 Relationship of the IS and immune checkpoints

We further analyzed the composition of immune markers in different IS subgroups (Figure S4). We then used ROC analysis to compare the sensitivity and specificity of the prognostic value of IS with immune checkpoints. IS showed better prognostic value than immune checkpoints for overall DFS (AUC: 0.618 vs. PD‐L1 0.588, PD‐1 0.508, CTLA‐4 0.454), 3‐year DFS (0.644 vs. 0.588, 0.593, 0.437) and 5‐year DFS (0.646 vs. 0.581, 0.489, 0.423), which similar results were observed for overall OS and 5‐year OS (Figure S4). With the same method, we compared the prognostic value of IS in HR positive subtypes. IS showed better prognostic value in the luminal A subtype than in the luminal B subtype (Figure S5).

### 3.5 Development of nomogram with IS

**FIGURE 5:**
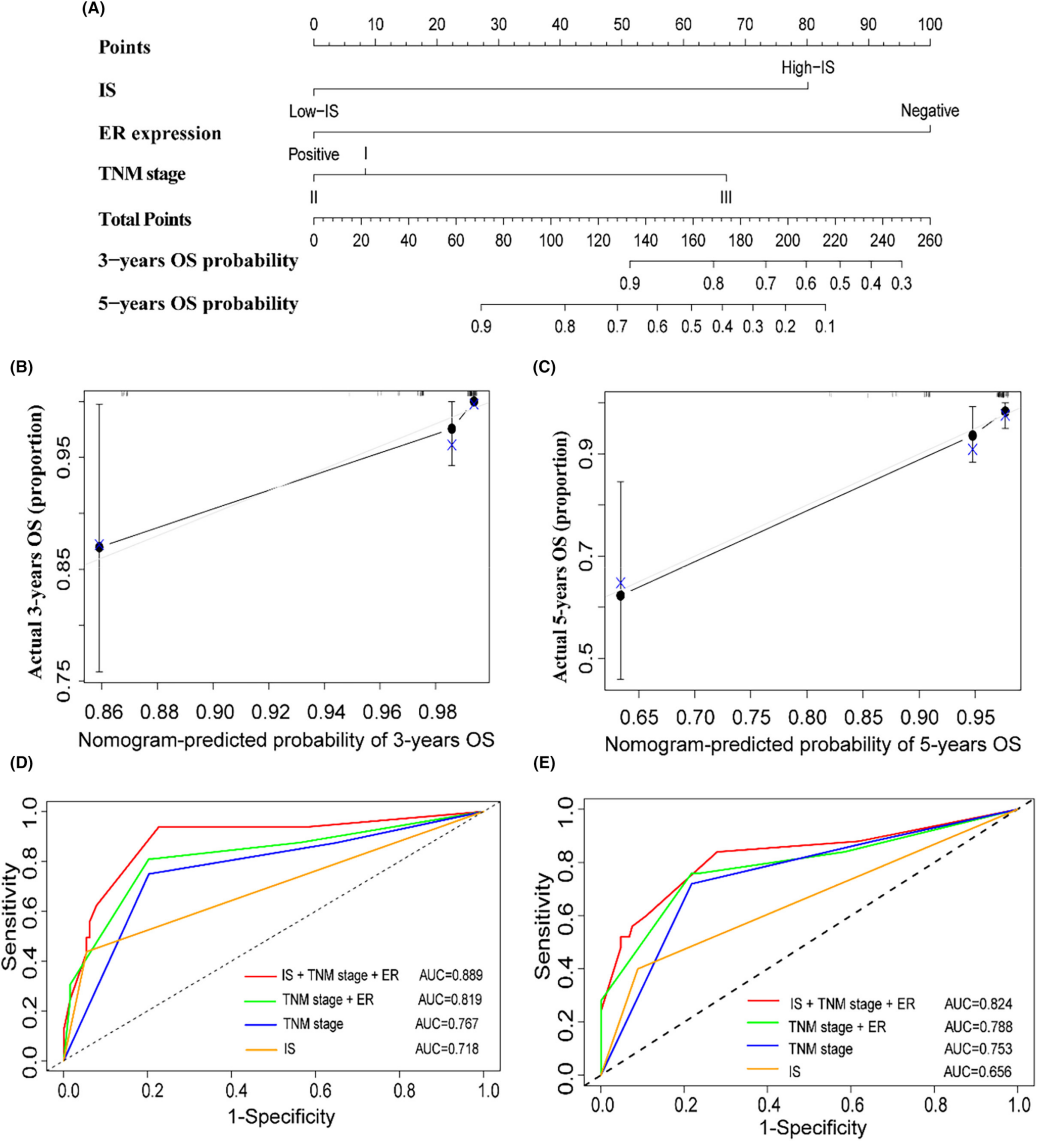
Nomogram and calibration plots for predicting 3‐year and 5‐year OS. (A) Nomogram A including IS, TNM stage and ER; (B, C) showed the calibration plots for predicting 3‐year and 5‐year OS in ILC patients; comparisons of the sensitivity and specificity for the prediction of 5‐year OS (D) and overall OS (E) by the combined IS, TNM stage and ER model, the TNM stage and ER model, the TNM stage alone model, and the IS alone model.

We constructed nomogram A combining IS, TNM stage, and ER to predict the 3 years and 5 years OS of ILC patients based on the multivariate Cox regression analysis (Figure 5A). In addition, we also constructed nomogram B using TNM stage and ER. The C‐index for the nomogram A to predict OS was 0.827 (95% CI 0.794–0.859), while the C‐index for the nomogram B was 0.786 (95% CI 0.75–0.822). The addition of IS to nomogram A slightly enhanced the accuracy compared with nomogram B. Subsequently, the calibration plot of nomogram A for the probability of 3 years or 5 years OS showed good agreement between the prediction by nomogram and actual observation for nomogram (Figure 5B, C). We used ROC analysis to compare the sensitivity and specificity of the prognostic score model with the TNM stage combined with ER, TNM stage, or IS alone model. Combination of the IS, TNM stage, and ER showed better prognostic value than the TNM stage and ER (AUC: 0.889 vs. 0.819), TNM stage alone (AUC: 0.889 vs. 0.767) for 5 years OS and, overall OS (AUC: 0.824 vs. 0.788 vs. 0.753) (Figure 5D, E).

### 3.6 ISm and association with prognosis in the METABRIC dataset

**FIGURE 6:**
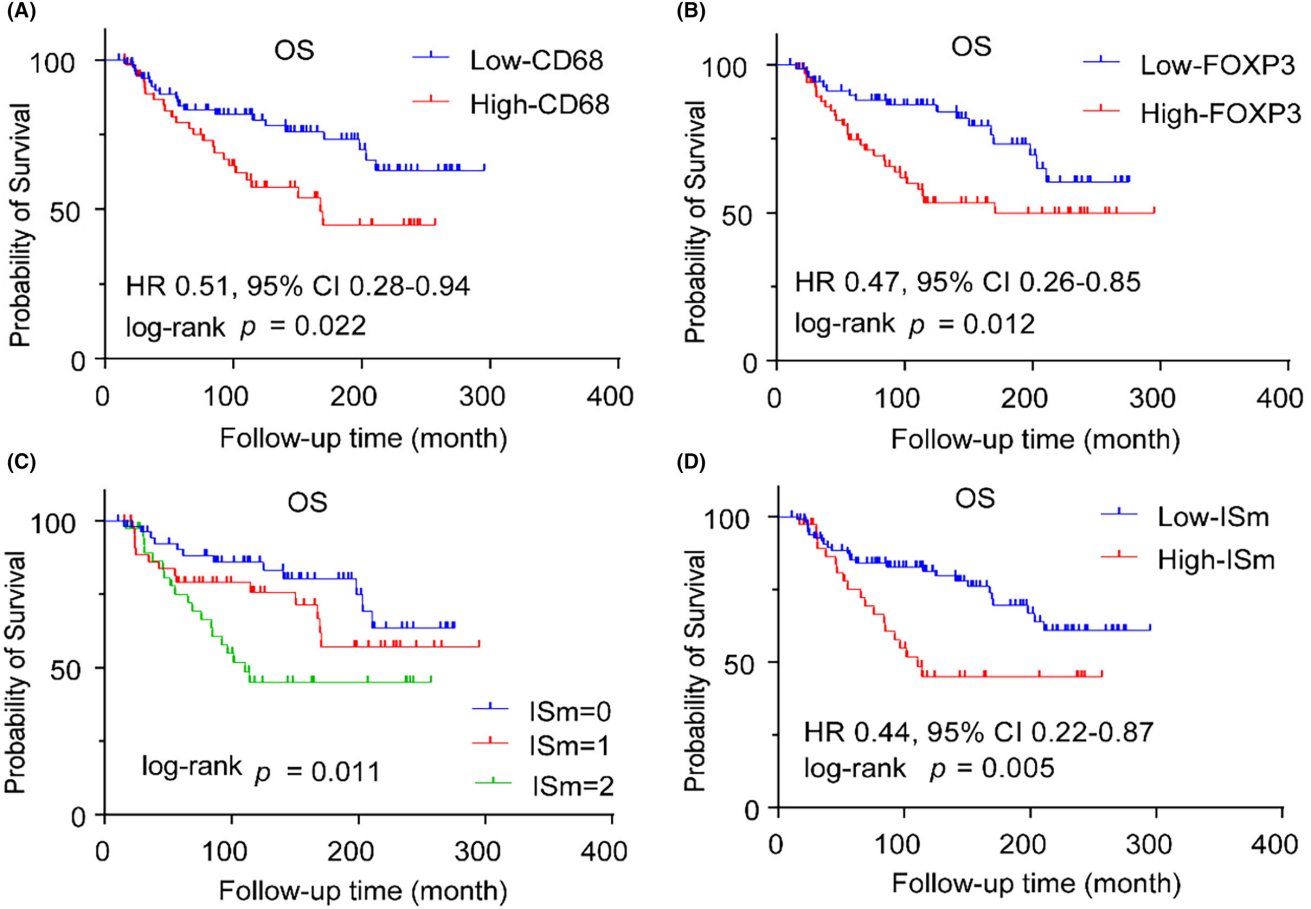
Kaplan–Meier curves for OS according to the different CD68^+^ macrophages, FOXP3^+^ T cells and immune score groups. Plots show the Kaplan–Meier curves of CD68 (A), FOXP3 (B) and different ISm subgroups (C). Patients were stratified in high and low ISm groups using the optimal cutoff value. Kaplan–Meier curves comparing 10‐year OS (C) and overall OS (D) in patients with high and low ISm groups.

We further explored the prognostic value of CD68 and FOXP3 and their ISm with the transcriptome data of ILC patients (*n* = 139) in the METABRIC database. The clinical characteristics of ILC patients in the METABRIC database are summarized in Table S2. To analyze the composition of immune cells in the METABRIC database, we used the Wilcoxon test to compare the relative proportions of 22 types of immune cells among different clinicopathological factors and ISm subgroups (Figure S6). We then explored the prognostic value of CD68^+^ macrophages and FOXP3^+^ T cells. Patients with low‐infiltrating CD68^+^ macrophages (*p* = 0.022) and FOXP3^+^ T cells (*p* = 0.012) had better OS than patients with high‐infiltrating (Figure 6A, B).

According to the ISm grouping method outlined above, 39.6%, 32.4%, and 28.1% of the patients were classified as ISm‐0, ISm‐1, and ISm‐2, respectively. We observed the same prognostic effect of ISm in METABRIC ILC cohort as IS in our own cohort. The Kaplan–Meier curves revealed three distinct patient groups with statistically significant differences in OS times (*p* = 0.011, Figure 6C). Patients with an ISm‐0 experienced the best outcome, while patients with an ISm‐2 had the worst outcome. Patients with a high degree of immune cell infiltration (ISm: 2) were assigned as the high‐ISm group, and patients with a low degree of immune cell infiltration (ISm: 0–1) were assigned as the low‐ISm group. Based on this, 71.9% (*n* = 100) patients were assigned to the low‐ISm group and 28.1% (*n* = 39) patients to the high‐ISm group. The patients with low‐ISm also had longer OS (HR 0.44, 95% CI 0.22–0.87, *p* = 0.005) than those with high‐ISm (Figure 6D). We also performed univariate and multivariate Cox regression analysis, which showed that the ISm remained significant for OS (Figure S7A,B). ISm also could be as a prognostic factor for 10‐year OS (AUC = 0.684) and overall OS (AUC = 0.605) in the METABRIC cohort (Figure S7C, D).

## 4 DISCUSSION

In the present study, we determined the density of nine immunological variables derived from six immune cells and three immune checkpoints using computational pathology analysis, and evaluated their prognostic value in ILC patients. Next, we developed an IS based on the density of CD68^+^ macrophages and FOXP3^+^ T cells, which was an important factor in predicting prognosis in ILC patients. Furthermore, we found that IS was a powerfully independent predictor of DFS and OS. Then, we developed a nomogram to predicted the 3 years and 5 years OS of ILC patients combining all independent variables IS, TNM stage, and ER. In addition, we further explored the prognostic role of CD68 and FOXP3 and their ISm in the METABRIC dataset, and our results demonstrated that the ISm was a valid prognostic immune‐related biomarker for ILC. To the best of our knowledge, this is the first study to simultaneously measure nine different immunologic variables derived from six immune cells and three immune checkpoints in the TME using computational pathology analysis, as well as to construct an IS for ILC, and to evaluate its prognostic significance.

Computational pathology analysis has recently produced encouraging results due to its quantitative, automated, and reproducible evaluation of whole‐slide sets.^30^ This permits automatic and accurate large‐scale analysis without subjective bias. In addition, algorithms based on “deep learning” neural networks have translated well to digital pathology, where they have demonstrated outstanding performance in tasks like tissue segmentation, prognostication, and computational TILs assessment.^11^, ^21^, ^31^ Particularly for IS, it demands accurate and quantitative evaluation of immune cells in the nonnecrotic invasive carcinoma area. However, due to factors of breast cancer, such as the spatial distribution of TILs, the tumor‐stroma ratio, histologic subtypes, TILs in ductal carcinomas in situ, and intra‐tumoral heterogeneity, which may increase the interobserver and intra‐observer variability of visual TIL assessments. Thus, it is necessary to deeply explore the immune markers properties of ILC using computational pathology analysis. In this study, we evaluated the density of six immune cells and three immune checkpoints through computational pathology analysis, which was developed based on the Xception model, achieved good performance in identifying TC and TAIC nuclei and accurate classification of cell types. Moreover, our digital pathology can obtain a large amount of quantitative information with high speed, which may quickly determine the IS of each patient.

Breast cancer was previously considered a relatively weakly immunogenic tumor compared to other tumor types. Recent evidence has suggested that TILs have prognostic and predictive capabilities for TNBC and HER2‐positive breast cancers.^32^, ^33^ However, the density of TILs and immune checkpoints in the tumor‐immune microenvironment of HR‐positive ILC is still unclear. Desmedt et al^34^ showed that TILs levels were statistically significantly lower in ILC compared with invasive ductal cancer, and high TILs levels were associated with worse prognosis in ILC. However, the ability to visual TILs assessment on the basis of H&E slides is highly subjective, less reproducible, and interobserver and intra‐observer variability. Furthermore, Pagès et al^12^ conducted a study comparing the differences between TILs and immunoscore, the results showed that immunoscore was highly objective, reproducible and had stronger ability of prognostic. Wang et al^35^ applied computational pathology to quantify the densities of CD3, CD8, and CD45RO in nasopharyngeal carcinoma, and then developed the IS based on the density of these three markers, and found that IS had an independent prognostic effect. In this study, we determined the density of six immune cells and three immune checkpoints using computational pathology analysis. In addition, immune cell markers CD68 and FOXP3 density was converted to a binary score to construct the IS, and found that IS has significant prognostic and predictive value in ILC. The results also showed that patients with high‐IS were associated with larger tumor size, lymph node metastasis, later stage, and faster proliferation, and were significantly associated with poorer DFS and OS.

Our study has shown that ILC patients with low density of CD4, CD8, CD20, CD56, CD68, and FOXP3 had significantly longer DFS and OS, in contrary to TILs in TNBC and HER2‐positive breast cancer.^32^, ^33^ Previous studies have shown that the level of TILs in ILC is significantly lower than that in invasive ductal carcinoma, and high level of TILs in ILC is associated with poor prognosis.^4^, ^34^ Studies have shown that the prognostic role of TIL subsets in breast cancer depends upon HR status and immune cells distribution.^35^, ^36^ Liu et al^37^ have reported that FOXP3+ regulatory TILs are a poor prognostic indicator in ER+ breast cancer, but a favorable prognostic factor in the HER2+/ER‐ subtype. Mahmoud et al^38^ found that higher numbers of CD68 macrophages were significantly associated with worse breast cancer‐specific survival and shorter disease‐free interval. Therefore, ILC patients with low density of immune cells have a better outcome, which may be related to the fact that ILC is mainly HR‐positive, and it may also be related to the underlying molecular features and biological mechanisms that are different between ILC and IDC.

Our study showed that CTLA‐4 was the highest expression among the densities of immune markers, and negatively correlated with other immune checkpoints and infiltrating immune cells. This suggested that ILC may be an immunosuppressive‐dominant tumor, which was consistent with a previous study.^2^ Moreover, our results demonstrated that the density of different immune checkpoints (PD‐1 and PD‐L1) could predict prognosis of ILC patients, and patients with high density had shorter DFS and OS. Conversely, low expression of CTLA‐4 was associated with poorer DFS and OS than high expression, which implied that anti‐CTLA‐4 therapy may be negatively correlated with response and survival in ILC patients. Recently, Santa‐Maria et al^39^ designed a single‐arm pilot research to determine the overall response‐rate (ORR) of durvalumab plus tremelimumab in metastatic ER‐positive or TNBC, and found that only three TNBC patients had a response (ORR = 17%). Tille et al^4^ reported that TILs were associated with larger tumors, lymph node involvement, HER2 amplification, and poor OS and iDFS was significantly associated with increasing TILs in ILC patients. The results of the two research were consistent with ours, that ILC was characterized by an immune‐suppressive phenotype with elevated expression of CTLA4, and may benefit less from anti‐CTLA4 therapy.

Currently, immunoscore has become a clinically useful prognostic marker in a variety of cancers, such as colorectal cancer, non‐small‐cell lung cancer and nasopharyngeal carcinoma.^11^, ^12^, ^40^ Until now, the prognostic significance of IS was unknown in ILC patients. In this study, ILC patients with low‐IS had significantly longer DFS and OS than those with high‐IS. Importantly, our results demonstrated that IS was an independent prognostic factor for DFS and OS. Furthermore, we further used ROC analysis to compare the prognostic value of IS with immune checkpoints. The results showed that IS had better prognostic value than immune checkpoints. Meanwhile, we also found IS had better prognostic value in Luminal A subtype than in Luminal B subtype. These results showed that IS was a promising prognostic classifier, which could be widely used to predict the prognosis of ILC patients. In addition, a prognostic score model combined the IS, TNM stage, and ER was constructed and had a better prognostic value than the TNM stage and ER, which could be an attractive tool to help in guiding treatment selection. The comprehensive immune score system can help to understand the immune state of tumors in individuals and improve the accuracy of TNM staging for predicting survival. Therefore, we believe it's more significant to identify IS for ILC patients based on the density of CD68 and FOXP3. We further explored the prognostic role of CD68 and FOXP3 and their ISm in the METABRIC dataset, and found that low proportion of CD68 and FOXP3 and their ISm were associated with longer OS, and ISm was also an independent prognostic factor for OS.

There were several limitations which should be considered while interpreting the study findings. Firstly, the density of immune cells detected by IHC, while the relative proportions of immune cells measured by transcriptome data, this difference may introduce bias in the analysis procedure. Secondly, immune markers were not assessed separately from the tumor and stroma within the invasive tumor's borders, as the distribution of different regions may affect the prognosis and treatment of ILC. Thirdly, this was a single‐center retrospective small‐sample study, should be further verified in a larger multicenter cohort.

## 5 CONCLUSIONS

Our study demonstrated that IS, which was based on the density of CD68 and FOXP3, was a promising immune‐related prognostic biomarker of ILC patients and might be an attractive option to help guide treatment selection.

## AUTHOR CONTRIBUTIONS

**Liye Wang:** Conceptualization (lead); data curation (lead); formal analysis (equal); methodology (lead); software (lead); writing – original draft (lead). **Peng Sun:** Data curation (equal); methodology (equal); software (supporting); writing – review and editing (equal). **Fei Xu:** Investigation (supporting); methodology (equal); supervision (equal); visualization (supporting); writing – review and editing (supporting). **Qiufan Zheng:** Data curation (supporting); methodology (equal); visualization (supporting). **Kuikui Jiang:** Data curation (supporting); software (supporting); validation (equal). **Ruoxi Hong:** Conceptualization (equal); data curation (equal); methodology (equal); resources (equal); supervision (lead); writing – review and editing (lead). **Shusen Wang:** Conceptualization (lead); data curation (equal); methodology (supporting); supervision (equal); writing – review and editing (supporting).

## FUNDING INFORMATION

The work was funded by the Sun Yat‐sen University Clinical Research 5010 Program (2017011), Natural Science Foundation of Guangdong Province (2023A1515030092), and National Natural Science Foundation of China (82372590).

## CONFLICT OF INTEREST STATEMENT

None declared.

## ETHICS STATEMENT

The study has been approved by the ethical committee of Sun Yat‐Sen University Cancer Center (B2021‐061). All procedures in this study were conducted in accordance with ethical principles.

## Supporting information


Figures S1–S7

Tables S1–S2

## Data Availability

The datasets used and analyzed during the current study are available from the corresponding author on reasonable request.

## References

[1] Mccart Reed AE , Kalinowski L , Simpson PT , et al. Invasive lobular carcinoma of the breast: the increasing importance of this special subtype. Breast Cancer Res. 2021;23(1):6. 33413533 10.1186/s13058-020-01384-6 PMC7792208

[2] Michaut M , Chin SF , Majewski I , et al. Integration of genomic, transcriptomic and proteomic data identifies two biologically distinct subtypes of invasive lobular breast cancer. Sci Rep. 2016;6:18517. 26729235 10.1038/srep18517 PMC4700448

[3] Desmedt C , Zoppoli G , Gundem G , et al. Genomic characterization of primary invasive lobular breast cancer. J Clin Oncol. 2016;34(16):1872‐1881. 26926684 10.1200/JCO.2015.64.0334

[4] Tille JC , Vieira AF , Saint‐Martin C , et al. Tumor‐infiltrating lymphocytes are associated with poor prognosis in invasive lobular breast carcinoma. Mod Pathol. 2020;33(11):2198‐2207. 32404955 10.1038/s41379-020-0561-9

[5] Ciriello G , Gatza ML , Beck AH , et al. Comprehensive molecular portraits of invasive lobular breast cancer. Cell. 2015;163(2):506‐519. 26451490 10.1016/j.cell.2015.09.033 PMC4603750

[6] Thompson ED , Taube JM , Asch‐Kendrick RJ , et al. PD‐L1 expression and the immune microenvironment in primary invasive lobular carcinomas of the breast. Mod Pathol. 2017;30(11):1551‐1560. 28731046 10.1038/modpathol.2017.79

[7] Arneth B . Tumor Microenvironment. Medicina (Kaunas). 2019;56(1):15. 31906017 10.3390/medicina56010015 PMC7023392

[8] Fridman WH , Pages F , Sautes‐Fridman C , et al. The immune contexture in human tumours: impact on clinical outcome. Nat Rev Cancer. 2012;12(4):298‐306. 22419253 10.1038/nrc3245

[9] Loi S , Drubay D , Adams S , et al. Tumor‐infiltrating lymphocytes and prognosis: a pooled individual patient analysis of early‐stage triple‐negative breast cancers. J Clin Oncol. 2019;37(7):559‐569. 30650045 10.1200/JCO.18.01010 PMC7010425

[10] Dieci MV , Conte P , Bisagni G , et al. Association of tumor‐infiltrating lymphocytes with distant disease‐free survival in the ShortHER randomized adjuvant trial for patients with early HER2+ breast cancer. Ann Oncol. 2019;30(3):418‐423. 30657852 10.1093/annonc/mdz007 PMC6442655

[11] Wang YQ , Chen L , Mao YP , et al. Prognostic value of immune score in nasopharyngeal carcinoma using digital pathology. J Immunother Cancer. 2020;8(2):e000334. 32690665 10.1136/jitc-2019-000334 PMC7371227

[12] Pages F , Mlecnik B , Marliot F , et al. International validation of the consensus Immunoscore for the classification of colon cancer: a prognostic and accuracy study. Lancet. 2018;391(10135):2128‐2139. 29754777 10.1016/S0140-6736(18)30789-X

[13] Gabrielson A , Wu Y , Wang H , et al. Intratumoral CD3 and CD8 T‐cell densities associated with relapse‐free survival in HCC. Cancer Immunol Res. 2016;4(5):419‐430. 26968206 10.1158/2326-6066.CIR-15-0110 PMC5303359

[14] Batlle E , Massague J . Transforming growth Factor‐beta signaling in immunity and cancer. Immunity. 2019;50(4):924‐940. 30995507 10.1016/j.immuni.2019.03.024 PMC7507121

[15] Wang X , Qi Y , Kong X , et al. Immunological therapy: a novel thriving area for triple‐negative breast cancer treatment. Cancer Lett. 2019;442:409‐428. 30419345 10.1016/j.canlet.2018.10.042

[16] Safonov A , Jiang T , Bianchini G , et al. Immune gene expression is associated with genomic aberrations in breast cancer. Cancer Res. 2017;77(12):3317‐3324. 28428277 10.1158/0008-5472.CAN-16-3478

[17] Giuliano AE , Edge SB , Hortobagyi GN . Eighth edition of the AJCC cancer staging manual: breast cancer. Ann Surg Oncol. 2018;25(7):1783‐1785. 29671136 10.1245/s10434-018-6486-6

[18] Curtis C , Shah SP , Chin SF , et al. The genomic and transcriptomic architecture of 2,000 breast tumours reveals novel subgroups. Nature. 2012;486(7403):346‐352. 22522925 10.1038/nature10983 PMC3440846

[19] Zhai Q , Fan J , Lin Q , et al. Tumor stromal type is associated with stromal PD‐L1 expression and predicts outcomes in breast cancer. PloS One. 2019;14(10):e0223325. 31584964 10.1371/journal.pone.0223325 PMC6777798

[20] Salgado R , Denkert C , Demaria S , et al. The evaluation of tumor‐infiltrating lymphocytes (TILs) in breast cancer: recommendations by an international TILs working group 2014. Ann Oncol. 2015;26(2):259‐271. 25214542 10.1093/annonc/mdu450 PMC6267863

[21] Sun P , He J , Chao X , et al. A computational tumor‐infiltrating lymphocyte assessment method comparable with visual reporting guidelines for triple‐negative breast cancer. EBioMedicine. 2021;70:103492. 34280779 10.1016/j.ebiom.2021.103492 PMC8318866

[22] Wang YQ , Zhang Y , Jiang W , et al. Development and validation of an immune checkpoint‐based signature to predict prognosis in nasopharyngeal carcinoma using computational pathology analysis. J Immunother Cancer. 2019;7(1):298. 31722750 10.1186/s40425-019-0752-4 PMC6854706

[23] Kumar N , Verma R , Anand D , et al. A multi‐organ nucleus segmentation challenge. IEEE Trans Med Imaging. 2020;39(5):1380‐1391. 31647422 10.1109/TMI.2019.2947628 PMC10439521

[24] Chollet F . Xception: deep learning with depthwise separable convolutions[C]// Xception: deep learning with depthwise separable convolutions. *Proceedings of the IEEE Conference on Computer Vision and Pattern Recognition* . 1251–1258.

[25] Tibshirani R . The lasso method for variable selection in the cox model. Stat Med. 1997;16(4):385‐395. 9044528 10.1002/(sici)1097-0258(19970228)16:4<385::aid-sim380>3.0.co;2-3

[26] Friedman J , Hastie T , Tibshirani R . Regularization paths for generalized linear models via coordinate descent. J Stat Softw. 2010;33(1):1‐22. 20808728 PMC2929880

[27] Kim YJ . Subverting the adaptive immune resistance mechanism to improve clinical responses to immune checkpoint blockade therapy. Onco Targets Ther. 2014;3(12):e954868. 10.4161/21624011.2014.954868 PMC4352965 25964860

[28] Camp RL , Dolled‐Filhart M , Rimm DL . X‐tile: a new bio‐informatics tool for biomarker assessment and outcome‐based cut‐point optimization. Clin Cancer Res. 2004;10(21):7252‐7259. 15534099 10.1158/1078-0432.CCR-04-0713

[29] Harrell FE Jr , Califf RM , Pryor DB , et al. Evaluating the yield of medical tests. Jama. 1982;247(18):2543‐2546. 7069920

[30] Amgad M , Stovgaard ES , Balslev E , et al. Report on computational assessment of tumor infiltrating lymphocytes from the international Immuno‐oncology biomarker working group. NPJ Breast Cancer. 2020;6:16. 32411818 10.1038/s41523-020-0154-2 PMC7217824

[31] Mobadersany P , Yousefi S , Amgad M , et al. Predicting cancer outcomes from histology and genomics using convolutional network. Proc Natl Acad Sci U S A. 2018;115(13):E2970‐E2979. 29531073 10.1073/pnas.1717139115 PMC5879673

[32] Pruneri G , Vingiani A , Bagnardi V , et al. Clinical validity of tumor‐infiltrating lymphocytes analysis in patients with triple‐negative breast cancer. Ann Oncol. 2016;27(2):249‐256. 26598540 10.1093/annonc/mdv571

[33] Luen SJ , Salgado R , Fox S , et al. Tumour‐infiltrating lymphocytes in advanced HER2‐positive breast cancer treated with pertuzumab or placebo in addition to trastuzumab and docetaxel: a retrospective analysis of the CLEOPATRA study. Lancet Oncol. 2017;18(1):52‐62. 27964843 10.1016/S1470-2045(16)30631-3 PMC5477653

[34] Desmedt C , Salgado R , Fornili M , et al. Immune infiltration in invasive lobular breast cancer. J Natl Cancer Inst. 2018;110(7):768‐776. 29471435 10.1093/jnci/djx268 PMC6037125

[35] Denkert C , Von Minckwitz G , Darb‐Esfahani S , et al. Tumour‐infiltrating lymphocytes and prognosis in different subtypes of breast cancer: a pooled analysis of 3771 patients treated with neoadjuvant therapy. Lancet Oncol. 2018;19(1):40‐50. 29233559 10.1016/S1470-2045(17)30904-X

[36] Baker K , Lachapelle J , Zlobec I , Bismar TA , Terracciano L , Foulkes WD . Prognostic significance of CD8+ T lymphocytes in breast cancer depends upon both oestrogen receptor status and histological grade. Histopathology. 2011;58(7):1107‐1116. 21707712 10.1111/j.1365-2559.2011.03846.x

[37] Liu S , Foulkes WD , Leung S , et al. Prognostic significance of FOXP3+ tumor‐infiltrating lymphocytes in breast cancer depends on estrogen receptor and human epidermal growth factor receptor‐2 expression status and concurrent cytotoxic T‐cell infiltration. Breast Cancer Res. 2014;16(5):432. 25193543 10.1186/s13058-014-0432-8 PMC4303113

[38] Mahmoud SM , Lee AH , Paish EC , et al. Tumour‐infiltrating macrophages and clinical outcome in breast cancer. J Clin Pathol. 2012;65(2):159‐163. 22049225 10.1136/jclinpath-2011-200355

[39] Santa‐Maria CA , Kato T , Park JH , et al. A pilot study of durvalumab and tremelimumab and immunogenomic dynamics in metastatic breast cancer. Oncotarget. 2018;9(27):18985‐18996. 29721177 10.18632/oncotarget.24867 PMC5922371

[40] Paulsen EE , Kilvaer T , Khanehkenari MR , et al. CD45RO(+) memory T lymphocytes–a candidate marker for TNM‐Immunoscore in squamous non‐small cell lung cancer. Neoplasia. 2015;17(11):839‐848. 26678911 10.1016/j.neo.2015.11.004 PMC4681889

