# Supplementary material for: Construction of immune score and its prognostic value in invasive lobular carcinoma of the breast using computational pathology analysis
Source: Cancer Med. 2023 Dec 27;13(1):e6896. doi: 10.1002/cam4.6896 (PMC10807639; doi:10.1002/cam4.6896)
Supplement: Supplementary file 1 — Figures S1–S7 [file CAM4-13-e6896-s001.docx]

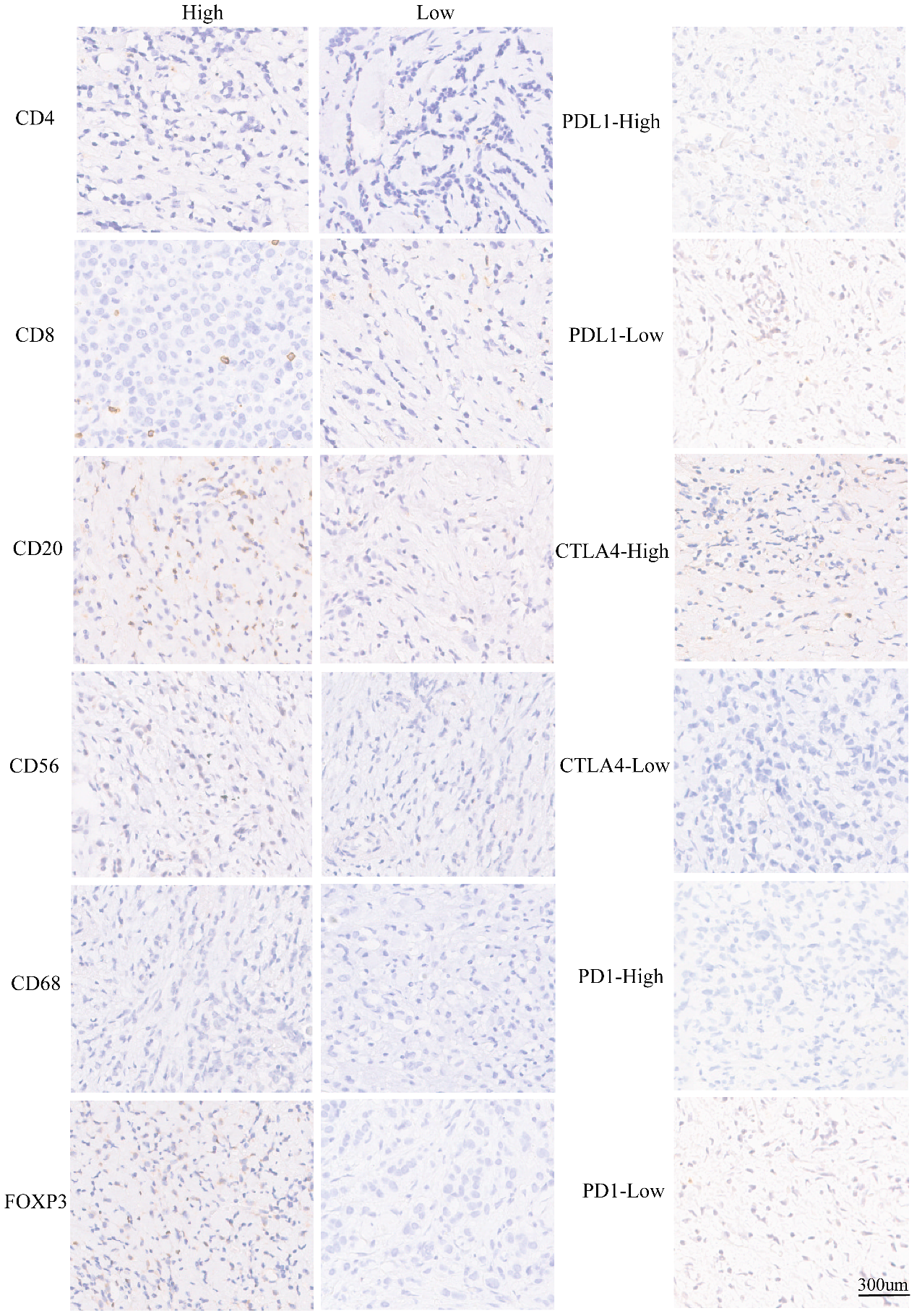


Figure S1 Representative images of tumor-associated immune cell markers and immune checkpoints from invasive lobular carcinoma determined by immunohistochemistry.


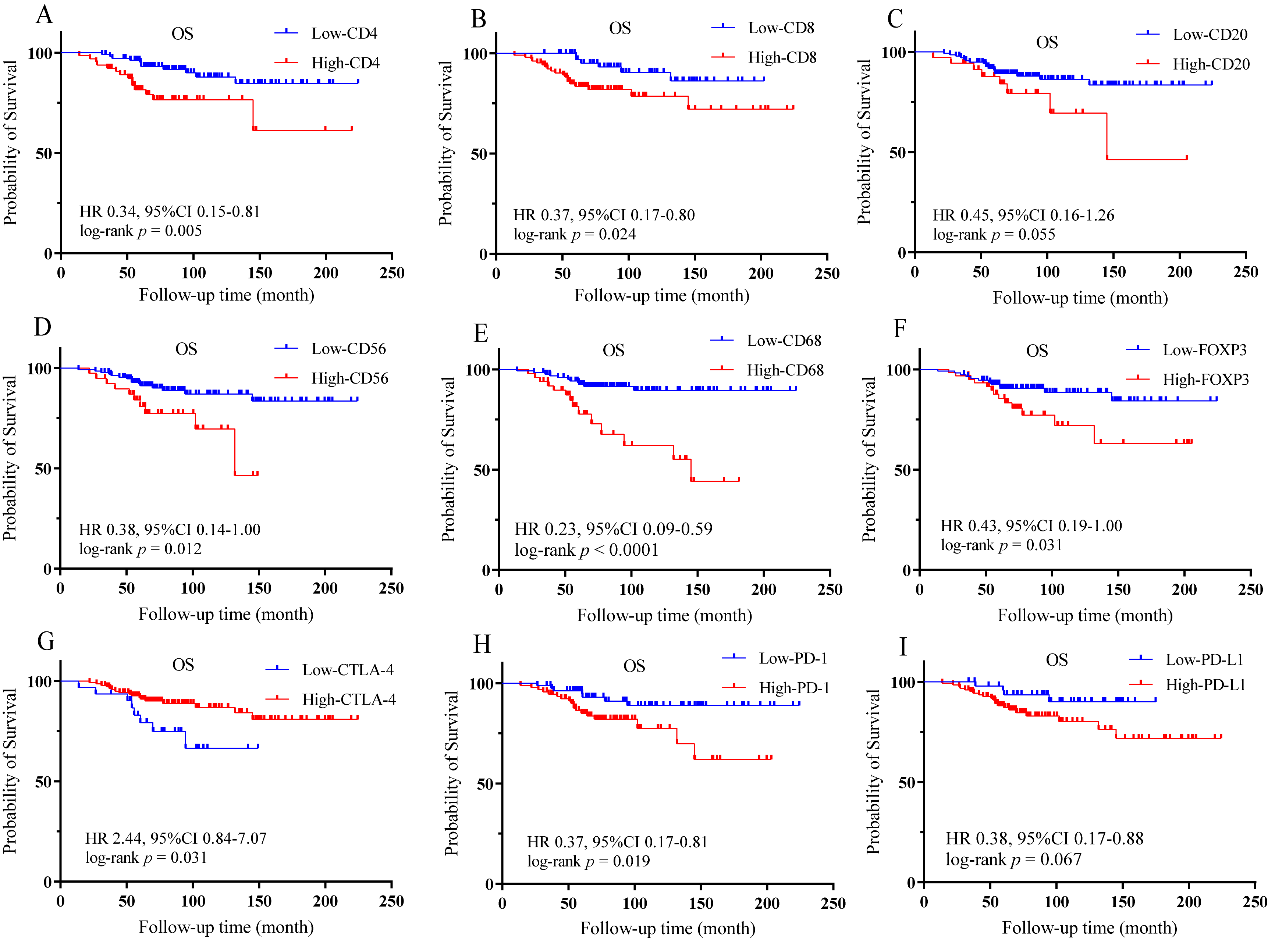


Figure S2 Kaplan-Meier curves for overall survival according to different immune markers density groups.

Plots show the Kaplan-Meier curves of CD4 (A), CD8 (B), CD20 (C), CD56 (D), CD68 (E), FOXP3 (F), CTLA-4 (G), PD-1 (H) and PD-L1 (I). Abbreviations: OS, overall survival.


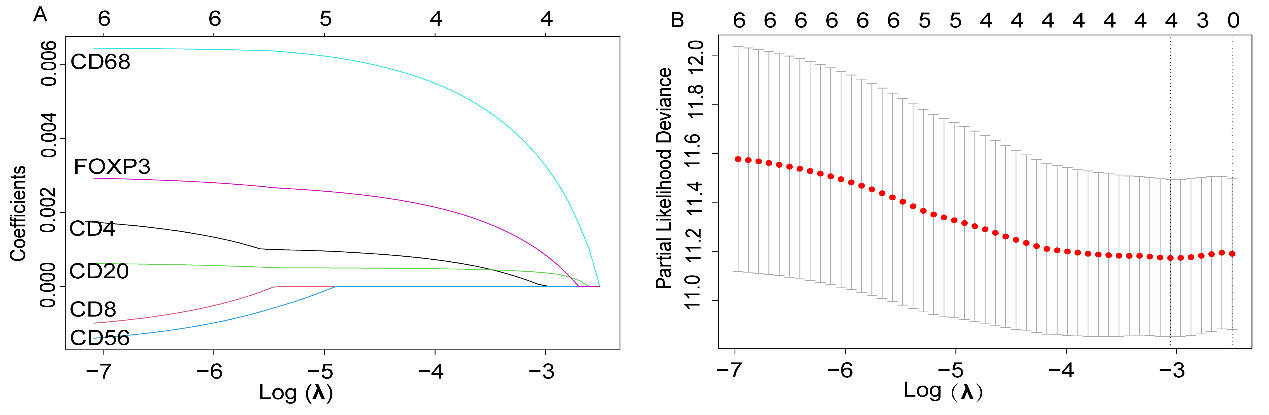


Figure S3 Immune cell markers were screened by LASSO Cox regression

(A) LASSO coefficient profiles of 6 immune cell marker features; (B) ten-time cross-validation for tuning parameter selection in the LASSO model.


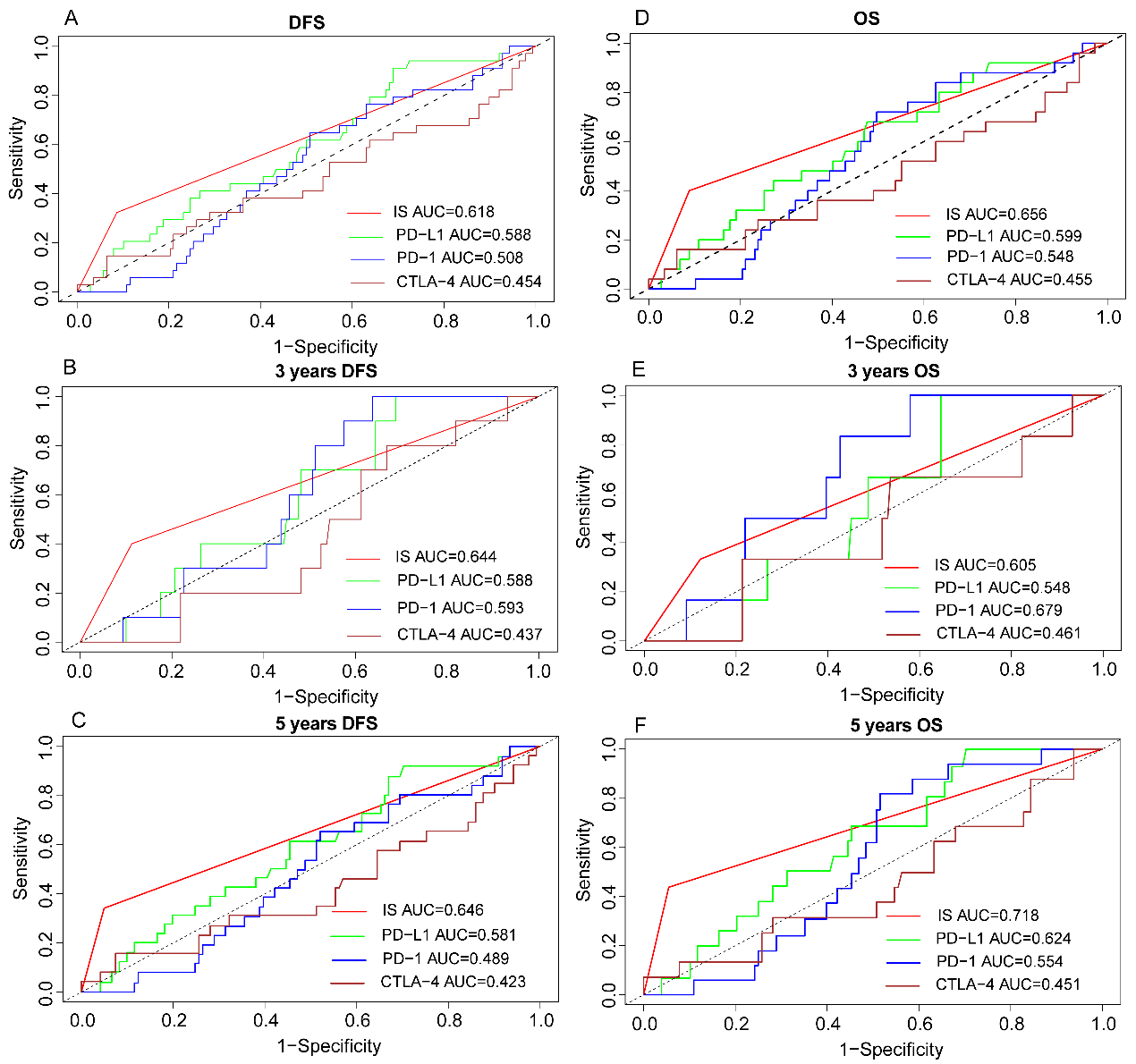


Figure S4 Prognostic role of IS and immune checkpoints in ILC

Comparison of the prognostic effects of IS and immune checkpoints in overall DFS (A), 3-year DFS (B), 5-year DFS (C) and overall OS (D), 3-year OS (E), 5-year OS (F). Abbreviations: IS, immune score; DFS, disease-free survival; OS, overall survival.


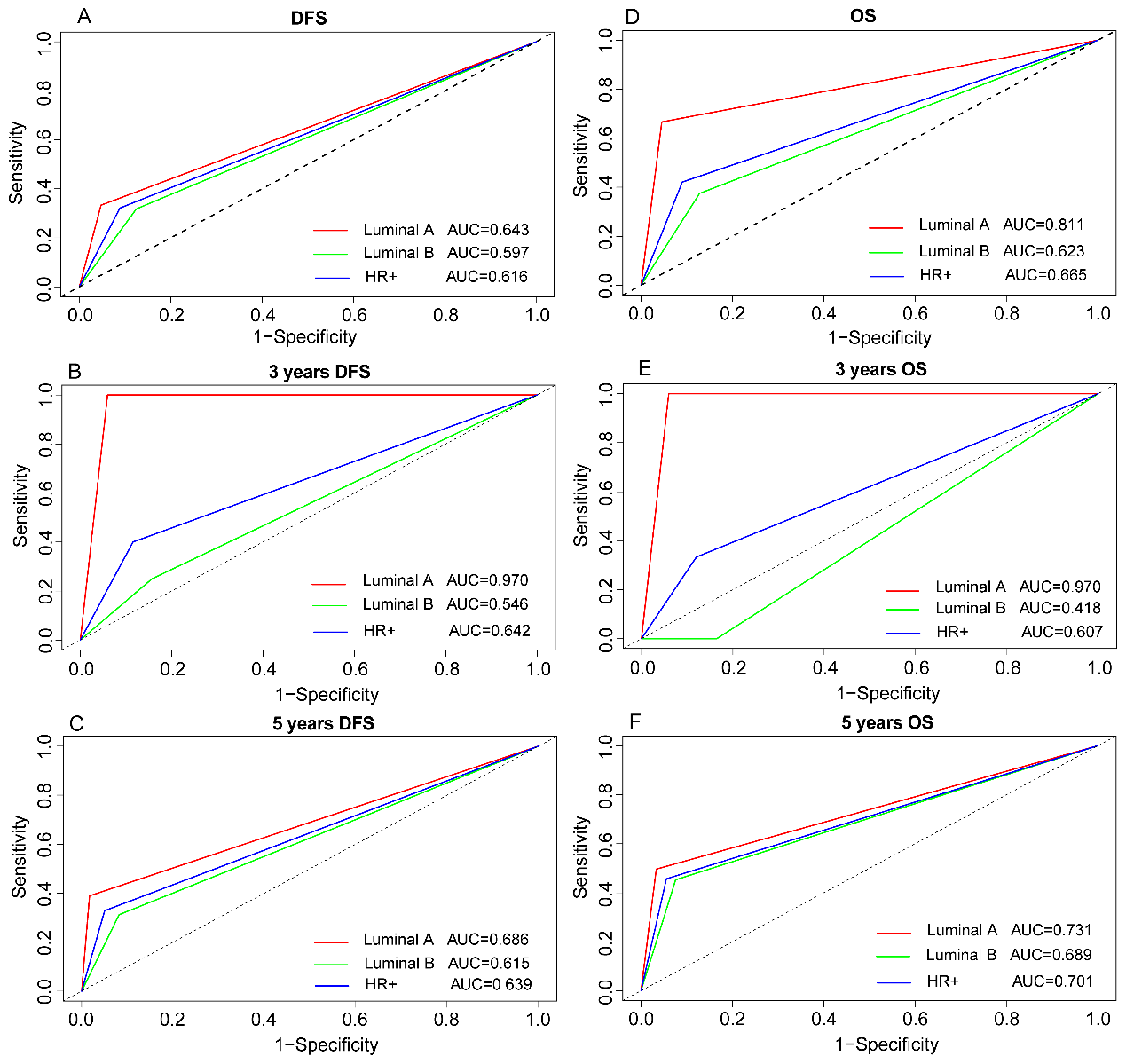


Figure S5 Prognostic role of IS in different molecular subtypes

Comparison of the prognostic effects of IS in different molecular subtypes for overall DFS (A), 3-year DFS (B), 5-year DFS (C) and overall OS (D), 3-year OS (E), 5-year OS (F). Abbreviations: IS, immune score; DFS, disease-free survival; OS, overall survival.


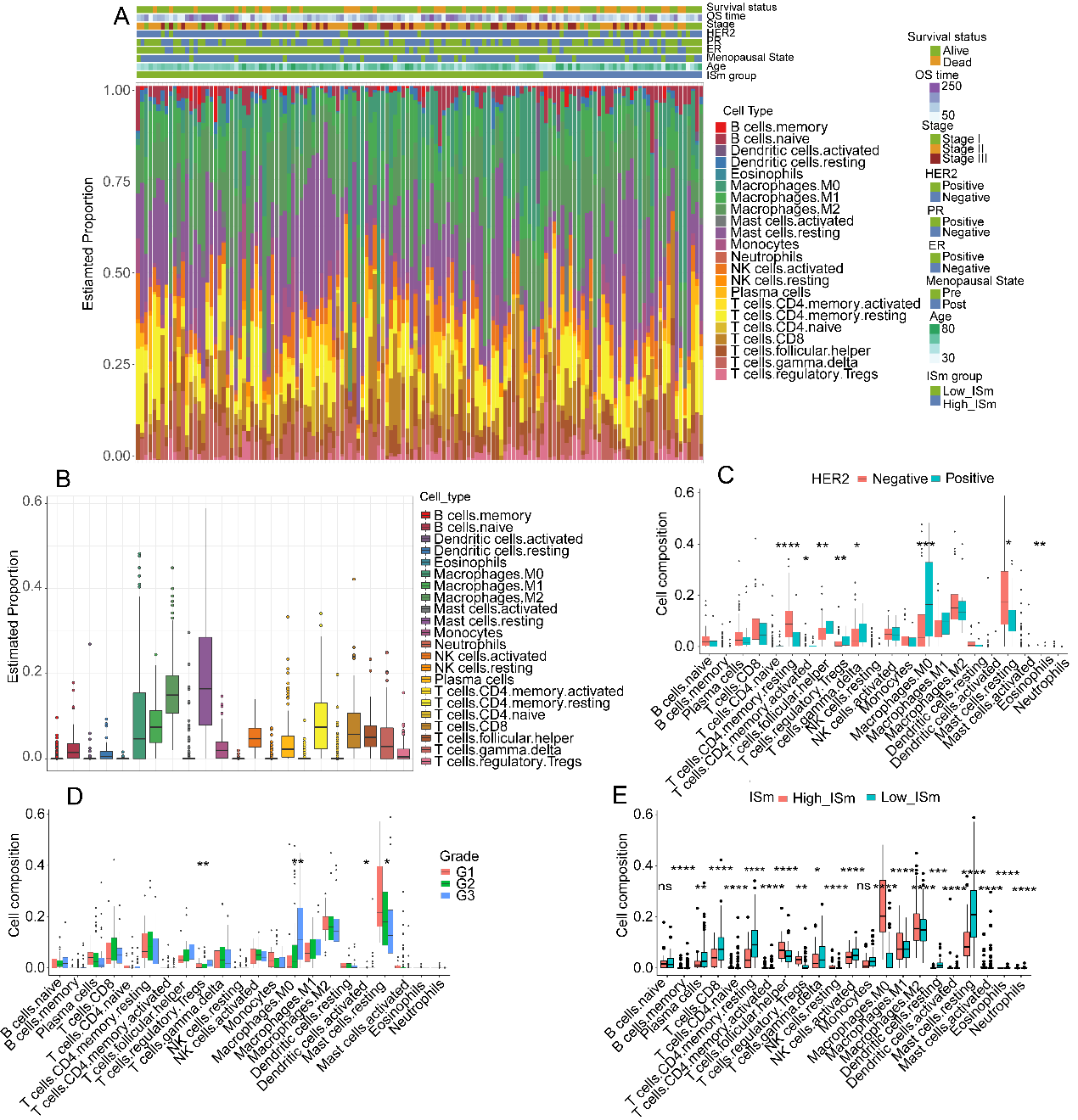


Figure S6 The landscape of the tumor microenvironment in ILC and the characteristics of different subgroups. (A) The IS grouping and proportions of TME cells for 139 patients in the METABRI cohort. Age, Menopausal state, ER, PR, HER2, Stage, OS time, and survival status are shown as patient annotations. (B) The proportions of TME cells in all samples. The proportions of TME cells in different HER2 (C), grade (D) and ISm (E) subgroups. The thick lines represent the median value. The bottom and top of the boxes are the 25th and 75th percentiles (interquartile range), respectively. The scattered dots represent the corresponding subgroups in the graph. Significant statistical differences between the two subgroups were assessed using the Mann–Whitney test (ns: not significant, **p* < 0.05, ** *p* < 0.01, *** *p* < 0.001, **** *p* < 0.0001). Abbreviations: ISm, immune score; ER, Estrogen receptor; PR, Progesterone receptor; HER2, human epidermal growth factor receptor 2.


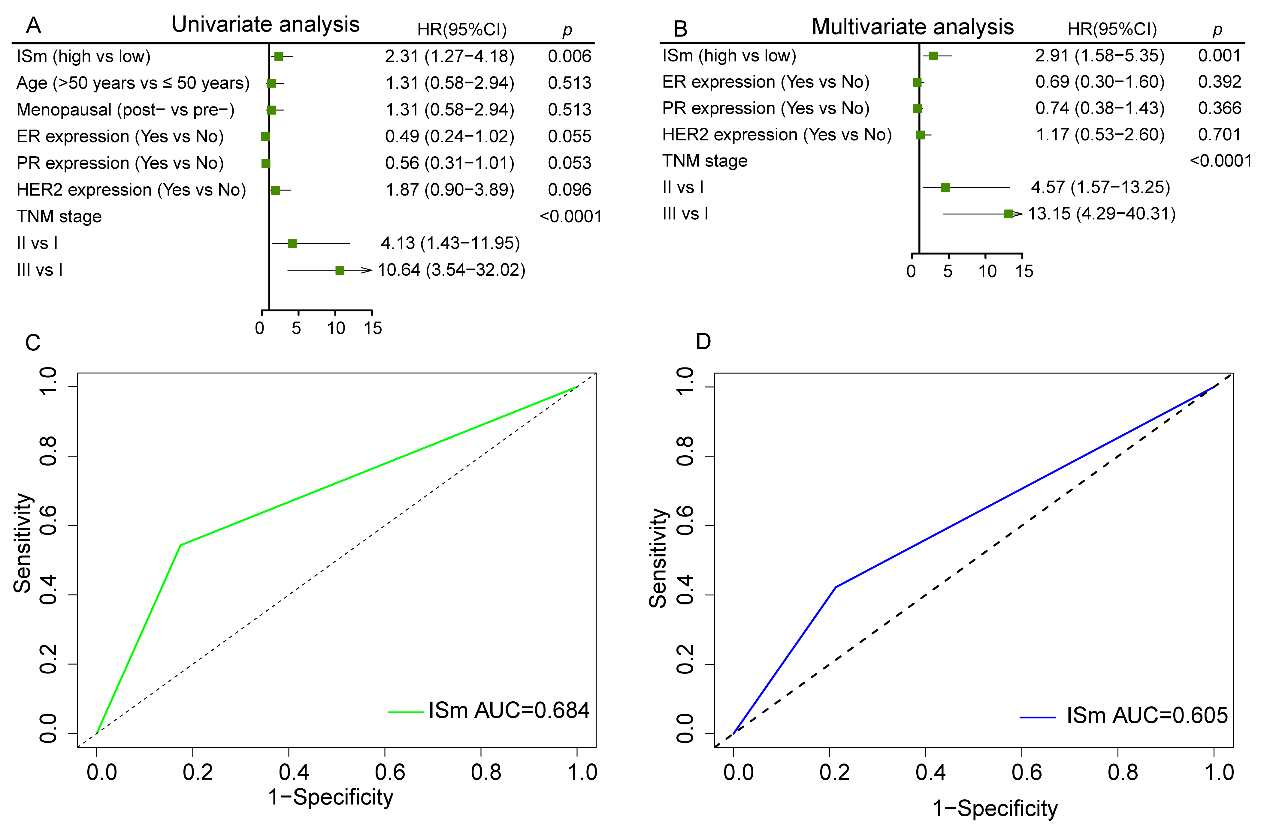


Figure S7 Prognostic role of ISm in the METABRIC cohort

Plots show univariate (A) and multivariate (B) analysis of OS; Receiver operating characteristics (ROC) curves for the prediction of 10-year OS (C) and overall OS (D).
