# Supplementary material for: Construction of immune score and its prognostic value in invasive lobular carcinoma of the breast using computational pathology analysis
Source: Cancer Med. 2023 Dec 27;13(1):e6896. doi: 10.1002/cam4.6896 (PMC10807639; doi:10.1002/cam4.6896)
Supplement: Supplementary file 2 — Tables S1–S2 [file CAM4-13-e6896-s002.docx]

Table S1 Expression levels of six infiltrating immune cells and three immune checkpoints in ILC

| Markers | Cut-off (cells/mm^2^) | Median (range) | Patients (n=172) |
| --- | --- | --- | --- |
| CD4 | 55.4 | 42.72 (3.10-427.27) |  |
| Low-CD4 |  | 27.20（3.10-54.33） | 107 (62.2) |
| High-CD4 |  | 241.33（55.40-427.27） | 65 (37.8) |
| CD8 | 43.29 | 56.35 (1.14-437.99) |  |
| Low-CD8 |  | 22.69（1.14-43.20） | 68 (39.5) |
| High-CD8 |  | 46.73（43.29-437.99） | 104 (60.5) |
| CD20 | 68.75 | 36.02 (4.39-1317.66) |  |
| Low-CD20 |  | 29.66（4.39-68.19） | 137 (79.7) |
| High-CD20 |  | 293.42（68.75-1317.66） | 35 (20.3) |
| CD56 | 16.42 | 8.89 (0.64-293.63) |  |
| Low-CD56 |  | 11.34（0.64-16.40） | 133 (77.3) |
| High-CD56 |  | 29.56（16.42-293.63） | 39 (22.7) |
| CD68 | 47.63 | 24.63 (1.06-259.09) |  |
| Low-CD68 |  | 34.04（1.06-47.60） | 123 (71.5) |
| High-CD68 |  | 118.62（47.63-259.09） | 49 (28.5) |
| FOXP3 | 69.31 | 45.17 (2.94-516.04) |  |
| Low-FOXP3 |  | 26.54（2.94-69.31） | 111 (64.5) |
| High-FOXP3 |  | 245.07（69.49-516.04） | 61 (35.5) |
| PD-1 | 29.64 | 34.55 (0.03-1268.16) |  |
| Low-PD-1 |  | 20.91（0.03-29.64） | 80 (46.5) |
| High-PD-1 |  | 88.56（30.58-1268.16） | 92 (53.5) |
| PD-L1 | 4.33 | 8.93 (0.15-295.97) |  |
| Low-PD-L1 |  | 3.88（0.15-4.33） | 49 (28.5) |
| High-PD-L1 |  | 9.16（4.34-295.97） | 123 (71.5) |
| CTLA-4 | 85.78 | 175.07 (6.58-905.92) |  |
| Low- CTLA-4 |  | 29.96（6.58-85.78） | 31 (18.0) |
| High- CTLA-4 |  | 319.99（86.39-905.92） | 141(82.0) |

Table S2 Clinicopathological characteristics of the patients stratified by ISm.

|  | All (n%) | Low (n%) | High (n%) | *p* value |
| --- | --- | --- | --- | --- |
| Total population | 139 (100) | 100 (71.9) | 39 (28.1) |  |
| Age |  |  |  | 0.296 |
| ≤50 years | 21 (15.1) | 13 (13.0) | 8 (20.5) |  |
| >50 years | 118 (84.9) | 87 (87.0) | 31 (79.5) |  |
| Menopausal status |  |  |  | 0.296 |
| Pre-menopausal | 21 (15.1) | 13 (13.0) | 8 (20.5) |  |
| Post-menopausal | 118 (84.9) | 87 (87.0) | 31 (79.5) |  |
| T stage |  |  |  | 0.697 |
| T1 | 52 (36.6) | 36（36.0） | 16（41.0） |  |
| T2-T3 | 87 (61.3) | 64 (64.0) | 23 (59.0) |  |
| N stage |  |  |  | 0.593 |
| N0 | 81 (58.5) | 56 (56.0) | 25 (64.1) |  |
| N1 | 29 (20.9) | 23 (23.0) | 6 (15.4) |  |
| N2-N3 | 29 (20.9) | 21 (21.0) | 8 (20.5) |  |
| TNM stage |  |  |  | 0.649 |
| I | 39 (28.1) | 26 (26.0) | 13 (33.3) |  |
| II | 66 (47.5) | 48 (48.0) | 18 (46.2) |  |
| III | 34 (24.5) | 26 (26.0) | 8 (20.5) |  |
| ER expression |  |  |  | 0.045 |
| Yes | 121 (87.1) | 91 (91.0) | 30 (76.9) |  |
| No | 18 (12.9) | 9 (9.0) | 9 (23.1) |  |
| PR expression |  |  |  | 0.851 |
| Yes | 72 (51.8) | 51 (51.0) | 21 (53.8) |  |
| No | 67 (48.2) | 49 (49.0) | 18 (46.2) |  |
| HER2 expression |  |  |  | 0.002 |
| Yes | 20 (14.4) | 8 (8.0) | 12 (30.8) |  |
| No | 119 (85.6) | 92 (92.0) | 27 (69.2) |  |
| Pam50 subtype |  |  |  | 0.034 |
| Luminal A | 61 (43.9) | 49 (49.0) | 12 (30.8) |  |
| Luminal B | 21 (15.1) | 13 (13.0) | 8 (20.5) |  |
| HER2-positive | 13 (9.4) | 5 (5.0) | 8 (20.5) |  |
| Basal | 11 (7.9) | 7 (7.0) | 4 (10.3) |  |
| Claudin-low | 10 (7.2) | 7 (7.0) | 3 (7.7) |  |
| Normal | 23 (16.5) | 19 (19.0) | 4 (10.3) |  |
| Chemotherapy |  |  |  | 0.149 |
| Yes | 16 (11.5) | 9 (9.0) | 7 (17.9) |  |
| No | 123 (88.5) | 91 (91.0) | 32 (82.1) |  |
| Radiotherapy |  |  |  | 1.000 |
| Yes | 75 (54.0) | 54 (54.0) | 21 (53.8) |  |
| No | 64 (46.0) | 46 (46.0) | 18 (46.2) |  |
| Endocrine therapy |  |  |  | 0.213 |
| Yes | 100 (71.9) | 75 (75.0) | 25 (64.1) |  |
| No | 39 (28.1) | 25 (25.0) | 14 (35.9) |  |
| Death |  |  |  | 0.015 |
| Yes | 45 (32.4) | 26 (26.0) | 19 (48.7) |  |
| No | 94 (67.6) | 74 (74.0) | 20 (51.3) |  |

Abbreviations: ISm, immune score; TNM, tumor-node-metastasis; ER, Estrogen receptor; PR, Progesterone receptor; HER2, human epidermal growth factor receptor 2.
